# Supplementary material for: Applications of artificial intelligence and urban innovation performance: A quasi-natural experiment based on the pilot zones for the innovative application of artificial intelligence
Source: PLoS One. 2025 Aug 12;20(8):e0329729. doi: 10.1371/journal.pone.0329729 (PMC12342255; doi:10.1371/journal.pone.0329729)
Supplement: S1 Appendix — (PDF) [file pone.0329729.s001.pdf]

The appendix is not presented in the main text.

Table 1. Specific Situations of the Construction of the Pilot Zones for the Innovative Application of Artificial Intelligence

Region	Year	Specific Implementation Situation
Pudong New Area, Shanghai	May 2019	On May 21st, China's first Pilot Zone for the Innovative Application of AI was launched and initiated for construction in Shanghai. With the "Mosu Space" as the core, Xuhui District rolled out an incubation space exceeding 60,000 square meters. An AI large language model science and innovation block was laid out along Longtai Road, driving the industrial upgrading of 1.6 million square meters of carrier space. Across the entire city, a large number of AI-related enterprises and scientific research forces have been gathered. Multiple innovative application scenarios have been established, and positive progress has been achieved in aspects such as AI technology research and development, industrial agglomeration, application expansion, and ecological construction.
Jinan-Qingdao	October 2019	The "Double Chain Leader" system has been implemented, and a "1+N" policy system has been established. Moreover, the "AI Spring City" empowerment action has been carried out. In 2024, the scale of the core AI industry reached 38.5 billion yuan, showing a year-on-year growth of 42%. In terms of industrial layout, a pattern of "one core, two wings, and multiple points" has been formed. Actively, a diversified innovation platform system has been constructed. Collaborations with universities, scientific research institutions, and enterprises have been carried out to establish research and development centers and so on, so as to promote technological research and development as well as application.
Shenzhen	October 2019	Build a "six-in-one" development system, and promote industrial development from multiple aspects such as computing power supply and technological innovation. Relying on key areas, establish "zero-rent" incubators, promote the construction of characteristic software renowned parks, set up industrial funds, and explore the linkage mechanisms of investment subsidies and investment loans. Invest up to 300 million yuan annually in basic research and technological research and development of AI, support the construction of source innovation centers for the domestic AI ecosystem and key laboratories, and encourage the open sourcing of software.
Beijing	February 2021	It puts forward goals such as reaching a scale of 300 billion yuan for the core AI industry by 2025. It proposes 16 key tasks focusing on technological research and development, industrial foundation, industrial phalanx, scenario construction, and innovation ecosystem. Moreover, it initiates the development of multiple standards, launches a "ten-billion-level" new quality productivity parallel fund, establishes the largest AI public computing power platform in Beijing and a leading one nationwide. The planned computing power scale exceeds 20,000 petaflops (P). Additionally, it constructs a data training base and opens up public data.
Binhai New Area, Tianjin	February 2021	Focusing on the coordinated development of the Beijing-Tianjin-Hebei region, we give full play to the policy advantages of the free trade zone, and promote breakthroughs in key areas such as intelligent manufacturing and smart ports, aiming to create industrial clusters such as "AI + information technology innovation". We have been advancing the construction in accordance with the "11251" strategy. As of now, we have deployed a computing power of 5000P, developed more than 20 large AI models, formed 432 AI application scenarios and benchmark projects. The industrial scale has exceeded 300 billion yuan, and there are more than 500 enterprises related to AI. In the assessment of the China Pilot Zone for AI Innovation and Application, it ranks the fourth.
Hangzhou	February 2021	Focusing on "computing power, algorithms, and data", Hangzhou will be the first to be built into a national source of AI technology innovation, an output hub of urban digital and intelligent governance

		solutions, a provider of intelligent manufacturing capabilities, an originator of data usage rules, and a main base for the development of the AI industry. Ten national landmark achievements will be achieved.
Guangzhou	February 2021	The public data open platform has gathered 66 open entities and 1,800 data sets, with a cumulative release of over 416 million pieces of data, covering multiple fields. A spatial layout of "one core and multiple points" has been constructed to promote industrial agglomeration and innovation, create industrial clusters, cultivate leading enterprises, and advance application demonstration projects.
Chengdu	February 2021	Based on the advantages of being an important hub of the Belt and Road Initiative, and by seizing the opportunities brought by the construction of the Chengdu-Chongqing Economic Circle, we take the empowerment of small and medium-sized enterprises by AI as a key approach, and focus on promoting development in industries such as healthcare and finance. Efforts should be made in three aspects: promoting the development of algorithms, driving the enhancement of the industrial level, and constructing an industrial ecosystem.
Nanjing	October 2022	Intensively formulate policies centered around the development of the AI industry, and implement five major actions, namely, strengthening the foundation of algorithm innovation, enhancing computing power support, aggregating and sharing data, enabling "AI +" application demonstrations, and upgrading industrial agglomeration. Focus on the "4266" industrial system, promote the integration of AI and various industries, and comprehensively improve the level of intelligence.
Changsha	October 2022	Based on the advantageous conditions such as a solid industrial foundation and strong infrastructure, the capacity and level of AI in empowering the real economy are comprehensively enhanced through such approaches as building a highland of computing power facilities, expanding the basic core industries, promoting breakthroughs in technological innovation, cultivating demonstration application scenarios, and creating samples of urban development.
Wuhan	October 2022	Accelerate the development and application of intelligent agents in basic general-purpose fields as well as vertical fields such as industry, healthcare, and education, and form a matrix of intelligent agent applications in Wuhan. Give full play to the role of the "Scene Office", create characteristic application scenarios, and plan the construction of AI industrial parks in the field of data services.
