# Supplementary material for: Applications of artificial intelligence and urban innovation performance: A quasi-natural experiment based on the pilot zones for the innovative application of artificial intelligence
Source: PLoS One. 2025 Aug 12;20(8):e0329729. doi: 10.1371/journal.pone.0329729 (PMC12342255; doi:10.1371/journal.pone.0329729)
Supplement: Attachment — Submitted filename: Response to Reviewers.pdf [file pone.0329729.s003.pdf]

Response to Reviewers：

We would like to extend our sincere gratitude to the Editor and all reviewers for their invaluable comments, which have significantly contributed to the improvement of the manuscript's quality. The reviewers' comments are listed in italicized text with specific questions numbered, our responses are presented in red font, and the revisions made to the manuscript are highlighted in yellow.

Editor:

1.*Please ensure that your manuscript meets PLOS ONE's style requirements, including those for file naming.*

Response:We have carefully reviewed our manuscript to ensure it meets PLOS ONE's style requirements, including those for file naming.

2.*PLOS requires an ORCID iD for the corresponding author in Editorial Manager on papers submitted after December 6th, 2016. Please ensure that you have an ORCID iD and that it is validated in Editorial Manager. To do this, go to 'Update my Information' (in the upper left-hand corner of the main menu), and click on the Fetch/Validate link next to the ORCID field. This will take you to the ORCID site and allow you to create a new iD or authenticate a pre-existing iD in Editorial Manager.*

Response:We confirm that the corresponding author has an ORCID iD, which has been validated in Editorial Manager.

3.*Please upload a copy of Supporting Information Figure/Table/etc. "Supporting information" which you refer to in your text on page 23 and 24.*

Response:We have uploaded the supporting information figure/table referred to in the text on pages 23 and 24. The file is labeled as "Supporting Information" and is included with this submission.

4.*Please review your reference list to ensure that it is complete and correct. If you have cited papers that have been retracted, please include the rationale for doing so in the manuscript text, or remove these references and replace them with relevant current references. Any changes to the reference list should be mentioned in the rebuttal letter that accompanies your revised manuscript. If you need to cite a retracted article, indicate the article's retracted status in the References list and also include a citation and full reference for the retraction notice.*

Response:We have meticulously reviewed the reference list to ensure its completeness and accuracy.

Reviewer 1:

Response: We sincerely appreciate your recognition of our research and your recommendation for accepting our manuscript. Minor revisions have been made to the study. Should any issues arise, we are prepared to make further corrections. Thank you very much.

Reviewer 2

1.*In line 330， "Currently, there are mainly four types of indicators for measuring urban innovation performance" ,but the author only wrote three in the narrative, do you need to change it?*

Response: Thank you for your suggestion. This is a part overlooked in our research

process. In the newly revised manuscript, we have supplemented the fourth point on the measurement method of urban innovation performance, which is specified in the revised manuscript with annotations.

2.*There is a problem with the city-fixed and time-fixed corner markers at the back of the formulae.*

Response: We greatly appreciate your meticulous reading. In accordance with this comment, we have revised the subscript part of the formula and made corresponding annotations.

3.*Parallel Trend Chart Pressure Lines for Benchmark Regression to be Adjusted.*

Response: Following a comprehensive data cleaning process, we have re-conducted the regression analysis. Additionally, the figures for the parallel trend test have been updated. Detailed revisions are clearly marked in the modified manuscript.

We have made every effort to improve the manuscript, including cleaning the data and making several revisions, none of which affect the content and framework of the article. Although the changes are not listed here, they are marked in yellow in the revised document. We sincerely appreciate the dedicated work of the editors and reviewers and hope that the corrections will be approved.
