# Supplementary material for: Applications of artificial intelligence and urban innovation performance: A quasi-natural experiment based on the pilot zones for the innovative application of artificial intelligence
Source: PLoS One. 2025 Aug 12;20(8):e0329729. doi: 10.1371/journal.pone.0329729 (PMC12342255; doi:10.1371/journal.pone.0329729)
Supplement: Attachment — Submitted filename: Response_to_Reviewers_auresp_2.pdf [file pone.0329729.s004.pdf]

Response to Reviewers：

We would like to express our sincere gratitude to the editor and all reviewers for their valuable comments, which have played an important role in improving the manuscript. In this response, the reviewers' comments are listed in italicized text with sequential numbering, our responses are presented in red font, and in the annotated manuscript, they are highlighted in red font.

Editor:

1.*Please review your reference list to ensure that it is complete and correct. If you have cited papers that have been retracted, please include the rationale for doing so in the manuscript text, or remove these references and replace them with relevant current references. Any changes to the reference list should be mentioned in the rebuttal letter that accompanies your revised manuscript. If you need to cite a retracted article, indicate the article's retracted status in the References list and also include a citation and full reference for the retraction notice.*

Response:Regarding the accuracy of the cited references, we have re-verified them by searching each reference one by one, and can confirm that there are no retracted articles in the reference list.

Reviewer 1:

1.*The author has carefully revised the manuscript and believes it is now polished and ready for publication, meeting all necessary standards and requirements.*

Response:Thank you for your recognition and affirmation of the manuscript revision! We have carefully improved the full text in accordance with the journal requirements and reviewers' comments to ensure that the manuscript quality meets the publication standards. We look forward to the smooth publication of this research achievement in your journal.

Reviewer 2:

1.*The author has made substantial revisions based on my suggestions, and I recommend acceptance of the manuscript. However, the author needs to incorporate all figures into the main text. Currently, all figures are provided only as supplementary materials and should be embedded within the article. Therefore, I recommend minor revision. Acceptance is recommended upon implementation of this amendment.*

Response:We hereby express our sincere gratitude for your acknowledgment of the manuscript revisions. We have noted the suggestion to integrate the figures and tables into the main text, and have completed this amendment by inserting them into the corresponding positions of the main text in order, ensuring that their formats comply with the journal's requirements. Thank you for your guidance and support.

We have standardized the decimal places throughout the article and supplemented the annotations on robust standard errors. Although these modifications are not listed individually here, they are marked in red in the revised document. We sincerely appreciate the dedicated efforts of the editors and reviewers and hope that these corrections will be approved.
