# Supplementary material for: Applications of artificial intelligence and urban innovation performance: A quasi-natural experiment based on the pilot zones for the innovative application of artificial intelligence
Source: PLoS One. 2025 Aug 12;20(8):e0329729. doi: 10.1371/journal.pone.0329729 (PMC12342255; doi:10.1371/journal.pone.0329729)
Supplement: Attachment — Submitted filename: Response_to_Reviewers_auresp_3.pdf [file pone.0329729.s005.pdf]

Response to Reviewers:

We would like to express our sincere gratitude to the editors and all reviewers for their valuable comments, which have played an important role in improving the quality of the manuscript. In this response, the editor's comments are listed in italics and numbered in order, while our responses are presented in red font. In the revised manuscript with track changes, the relevant paragraphs are highlighted in red font.

Additional Editor:

1.Please carefully check the formula spelling and instruction.

Response: Thank you very much for your valuable suggestion. We have compared the symbols, variable names, and expressions in the formula line by line and sentence by sentence to ensure that the formula is accurate. We have reorganized the formula explanation section and corrected any errors, as detailed below:

4.1 Model Construction

4.1.1 Multi-period DID Model

In the process of promoting the establishment of pilot zones for the innovative application of AI, various regions have focused mainly on the deep integration of AI and the real economy. The establishment of pilot zones for the innovative application of AI will lead not only to differences in innovation performance between pilot cities and non-pilot cities before and after the implementation of the policy but also to systematic differences in the individual characteristics of pilot cities and non-pilot cities, resulting in differences in urban innovation performance between pilot and nonpilot cities at the same point in time. Therefore, by establishing pilot zones for the innovative application of AI as a quasi-natural experiment, a multi-time-point DID model is constructed to evaluate the impact of AI applications on urban innovation performance. Accordingly, this study regards the pilot cities as the treatment group and the non-pilot cities as the control group and constructs the following multi-time-point DID model:

$$UIP_{it}\begin{cases} Iquality_{it} \\ Iquantity_{it} \end{cases} = \alpha_0 + \alpha_1 AI\_Policy_{it} + \alpha_2 \sum Controls_{it} + \gamma_i + \delta_t + \varepsilon_{it} \quad (1)$$

where *i* and *t* represent the city and year, respectively. The explained variable UIP_{it} is the performance of urban innovation, which is manifested as the innovation index of city *i* in year *t*, and

$Iquality_{it}$ and $Iquantity_{it}$ represent the quality of urban innovation and the quantity of urban innovation, respectively. AI_Policy_{it} is the establishment of the pilot zone for the innovative application of AI, which is the core explanatory variable of this study; $\sum Controls_{it}$ is the set of control variables; γ_i is the urban fixed effect; δ_t is the time fixed effect; and ε_{it} is the random disturbance term. The estimated coefficient measures the average difference in urban innovation performance before and after the establishment of the Pilot Zone for the Innovative Application of AI.

4.1.2 Mediating Effect Model

According to previous research, the level of human capital (Lhuca) and absorptive capacity (Cabsorp) are important channels through which AI promotes urban innovation. A mediating effect model was constructed to test this impact pathway.

$$X_{it} = \beta_0 + \beta_1 AI\_Policy_{it} + \beta_2 \sum Conlrols_{it} + \gamma_i + \delta_t + \varepsilon_{it} \quad (2)$$

$$UIP_{it}\begin{cases} Iquality_{it} \\ Iquantity_{it} \end{cases} = \gamma_0 + \gamma_1 AI\_Policy_{it} + \gamma_2 X_{it} + \gamma_3 \sum Controls_{it} + \gamma_i + \delta_t + \varepsilon_{it} \quad (3)$$

Among these variables, X_{it} serves as the mediating variable, which in this study represents the level of human capital and absorption capacity. β_1 measures the impact of AI applications on the mediating variable, and γ_1 represents the impact of AI applications on urban innovation performance after taking the mediating variable into account.

4.1.3 Moderating Effect Model

The empirical results presented earlier confirm that the degree of government intervention weakens the promoting effect of AI applications on urban innovation performance. To test this hypothesis, the following model was constructed:

$$UIP_{it}\begin{cases} Iquality_{it} \\ Iquantity_{it} \end{cases} = \rho_0 + \rho_1 AI\_Policy_{it} + \rho_2 AI\_Policy_{it} \times Dinter_{it} + \rho_3 \sum Control$$

$$+\gamma_i + \delta_i + \varepsilon_{it} \quad (4)$$

Among them, $AI\_Policy_{it} \times Dinter_{it}$ served as the moderating variable, and ρ_2 moderated the intervention effect.

2. The author need to polish the language.

Response: We thank you for your valuable suggestions. We made detailed modifications to the language of the manuscript according to your request of " the author need to polish the language ". At the same time, in order to better meet the submission requirements of the journal, we used Wiley language services to modify the English

language of the manuscript. The following is a proof of use of Wiley language services, as detailed below:

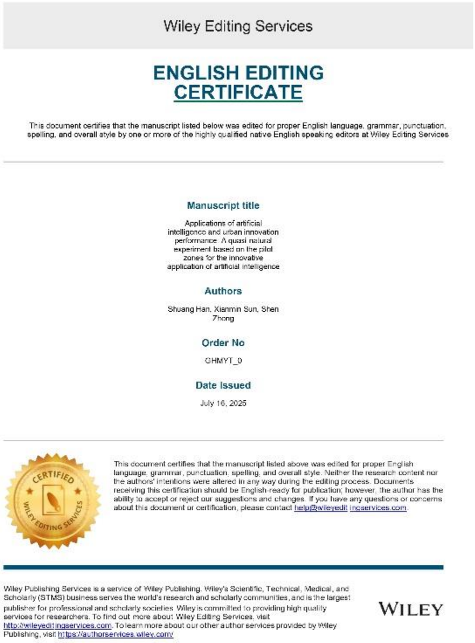

Wiley Editing Services

ENGLISH EDITING CERTIFICATE

This document certifies that the manuscript listed below was edited for proper English language, grammar, punctuation, spelling, and overall style by one or more of the highly qualified native English speaking editors at Wiley Editing Services

Manuscript title

Applications of artificial intelligence and urban innovation performance: A quasi natural experiment based on the pilot zones for the innovative application of artificial intelligence

Authors

Shuang Han, Xianmin Sun, Shen Zhong

Order No

GHMYT_0

Date Issued

July 16, 2025

This document certifies that the manuscript listed above was edited for proper English language, grammar, punctuation, spelling, and overall style. Neither the research content nor the authors' intentions were altered in any way during the editing process. Documents receiving this certification should be English-ready for publication; however, the author has the ability to accept or reject our suggestions and changes. If you have any questions or concerns about this document or certification, please contact help@wileyedit ingservices.com.

Wiley Publishing Services is a service of Wiley Publishing. Wiley's Scientific, Technical, Medical, and Scholarly (STMS) business serves the world's research and scholarly communities, and is the largest publisher for professional and scholarly societies. Wiley is committed to providing high quality services for researchers. To find out more about Wiley Editing Services, visit http://wileyeditingservices.com. To learn more about our other author services provided by Wiley Publishing, visit https://authorservices.wiley.com/

WILEY

Dear editor and reviewers,

In order to further meet the stringent publication requirements, we meticulously reviewed every word within the article, paying close attention to aspects such as punctuation and typography, thereby ensuring the academic rigor of the manuscript. Additionally, we diligently verified that each reference was cited accurately and in accordance with the prescribed citation format.

We have made every effort to improve the manuscript and have made some revisions. The revisions to the manuscript are mainly based on the comments provided. These revisions will further enhance the readability and presentation of the article. We sincerely thank the editors/reviewers for their hard work and hope that these revisions will be accepted. Thank you again for your valuable comments and suggestions.

You made my day!

Kind regards,

Authors
